# The Role of Peer Support Education Model in Management of Glucose and Lipid Levels in Patients with Type 2 Diabetes Mellitus in Chinese Adults

**DOI:** 10.1155/2019/5634030

**Published:** 2019-11-22

**Authors:** Xin Zhao, Xiaofeng Yu, Xiaomei Zhang

**Affiliations:** Endocrinology Department, Peking University International Hospital, Beijing, China

## Abstract

**Aims:**

To investigate the effect of peer support education model on the levels of glucose and lipids in patients with type 2 diabetes mellitus in China.

**Methods:**

120 type 2 diabetes mellitus patients (T2DM) were assigned to two groups randomly from December 2016 to October 2017. Group 1 was trained on basic diabetic medical knowledge by a professional medical staff. After 8 weeks of studying, these specially trained patients then trained the patients of Group 2.

**Results:**

It was found that after 3 months of intervention, the levels of fasting blood glucose, triglycerides, glycosylated hemoglobin, total cholesterol, and low-density lipoprotein cholesterol were significantly decreased for the two groups (*p* < 0.05). However, with the prolongation of time, there were no significant changes in the two groups in the above indices (*p* > 0.05) after 6 months, and the result was the same after strafing sex, age, and education level. Meanwhile, there was no significant difference in decreasing glucose and lipid level between the two groups' participants (*p* > 0.05).

**Conclusion:**

It was found that both the peer support education model and medical staff education model have a better short-term effect on blood glucose and lipid levels in patients with type 2 diabetes. As there is no difference of effect between the two methods of training, the peer support education model can be widely used in diabetes education.

## 1. Introduction

Due to changes in lifestyle and the acceleration of social aging, the incidence of diabetes worldwide increases year by year. Faced with the rise in the number of diabetic patients, it is of utmost importance to educate the public about the prevention and treatment of the disease, including the required change of lifestyle and dietary behavior of patients [1]. Fisher et al. claimed that “A diabetic patient spends only an average of six hours a year with their doctor, with the remaining approximate 8760 hours left to deal with the disease themselves.” [2]. Therefore, self-management plays a key role in the treatment of diabetes. Currently, health education mainly depends on the medical staff, but due to limited manpower and time constraints, the requirements to educate the evergrowing diabetic population are not met. Therefore, the need for a peer support education model for diabetic patients is extremely important. Peer support education is also known as peer counseling or peer education. It refers to a form of education in which peers with same or similar backgrounds and experiences share their experiences and learn from each other. In this scenario, it would result in a transfer of disease-related knowledge and skills in order to achieve the goal of health education.

Studies have confirmed that the peer support education model can aid in preventing and managing diabetes by improving the patient's lifestyle. It can help chronic patients by increasing their enthusiasm and compliance, aiding in self-management of the disease. The added benefit of the peer support education model, which can be conducted by patients themselves, can share the workload and burden of the medical staff. It also achieves better results than the sole intervention of a medical staff [3]. In recent years, the peer support education's positive influence on the self-management ability and blood glucose control levels of patients suffering from type 2 diabetes mellitus (T2DM) has been widely recognized. However, there are limited reports about whether there is a difference in the control of blood glucose levels between the peer support education and medical staff education. The purpose of this paper is to compare whether there is a difference between peer support education and education of medical staff, on the basis of blood glucose levels, blood pressure, and other metabolic indicators of patients suffering from T2DM. We aimed to evaluate whether peer education support can beneficially aid patients with T2DM.

## 2. Methodology

### 2.1. Study Design

The design for this study was approved by the Ethics Committee of the Peking International Hospital. The duration of the conducted study was from December 2016 to October 2017. 120 patients with T2DM were selected from the outpatient clinic of Peking University International Hospital, which included 65 males and 55 females. Using a random number table method, the participants were randomly assigned to two groups. Group 1 received training from professional medics and nursing staff. Group 2 received peer support education. Training courses were held once a week, an hour at a time, with a total of 8 courses over a duration of 2 months. The same training content about diabetes was given to the two groups which included diet, exercise, blood glucose monitoring, drug use, health debugging, problem solving, risk-factor control, and complication prevention. The program duration lasted for 6 months. The inclusion criteria of participants were (1) T2DM diagnostic criteria based on the World Health Organization (WHO) guidelines [4], (2) 18 to 70 years old, (3) glycosylated hemoglobin < 8.5%, and (4) willingness to participate in diabetes peer group activities and first time participation in peer support group activities. Exclusion criteria of participants were consciousness disorders, patients with mental problems, inadequate expressiveness, and any disease that the researchers considered would interfere with participation in the study or assessment. General details of the subjects were recorded, including height, weight, educational level, and disease duration before and after intervention. Meanwhile, both systolic and diastolic blood pressure (SBP and DBP) were measured and recorded. According to the weight and height of the individual, the body mass index (BMI) was calculated using the formula: BMI = weight/height^2^ [2].

### 2.2. Research Methods

#### 2.2.1. Training from Professional Staff and Peer Support Education Method

Group 1 received training courses which included basic medical knowledge and management skills of diabetes. The topics included diet, sports, monitoring, medicine, health adjustment, problem solving, risk-factor control, and prevention of complications.

Group 1 subjects mastered the specific operation steps of peer education. Courses were conducted once a week for an hour each time, resulting in a total of 8 courses over 2 months. The training methods included group teaching, group sharing, skill training, and simulation exercises. The peer group leader and the diabetes health education nurses worked out a research object activity mode for group 2. Subjects of group 1 regularly organized group activities, set up peer education groups on “We Chat,” and integrated online and offline activities. Subjects of group 1 provided lessons on self-management, shared the skills they learnt, provided emotional support, and gave daily reminders of visits and reviews to the participants of group 2. This was overseen by the medical staff to prevent misinformation. The peer group leaders regularly followed up to make sure all the participants obtained relevant information. All subjects had no adjustments to their hypoglycemic medication before and after the intervention. Meanwhile, all the participants were tested on their theoretical knowledge of diabetes and received the scale evaluation including Self-rating Depression Scale (SDS), WHO-5 Happiness Scale, Diabetes-Specific Quality of Life Scale (DSQOLS),Summary of Diabetes Self-Care Activities(SDSCA) including diet, sports, monitoring, medicine, solving problem, health adjustment, and control of risk factor.

#### 2.2.2. Medical Tests

Medical tests were conducted at three intervals to assess the patient's condition: before the study, 3 months and 6 months after the intervention. 10 ml of venous blood was collected after the subjects fasted for 8 hours. The biochemical indicators tested were fasting blood glucose (FPG), glycosylated hemoglobin (HbA1c), cholesterol (TC), low-density lipoprotein cholesterol (LDL-C), high-density lipoprotein cholesterol (HDL-C), triglyceride (TG), urea nitrogen (BUN), uric acid (UA), and serum creatinine (sCr). Based on serum creatinine levels, the glomerular filtration rate was calculated according to the CKD-EPI-ASIA equation as follows [5].

Males:

(1) $$\begin{array}{c} \text{sCr}<0.9\text{mg}/\text{dl}:\text{eGFR CKD}‐\text{EPI}‐\text{ASIA}*=141\times {\left(\text{sCr}/0.9\right)}^{-0.411}\times 0.993^{\text{age}}\times 1.057,\\ \text{sCr}>0.9\text{mg}/\text{dl}:\text{eGFR CKD}‐\text{EPI}‐\text{ASIA}=141\times {\left(\text{sCr}/0.9\right)}^{-1.209}\times 0.993^{\text{age}}\times 1.057. \end{array}$$

Females:

(2) $$\begin{array}{c} \text{sCr}<0.7\text{mg}/\text{dl}:\text{eGFR CKD}‐\text{EPI}‐\text{ASIA}=141\times {\left(\text{sCr}/0.7\right)}^{-0.329}\times 0.993^{\text{age}}\times 1.049,\\ \text{SCr}>0.7\text{mg}/\text{dl}:\text{eGFR CKD}‐\text{EPI}‐\text{ASIA}=141\times {\left(\text{sCr}/0.7\right)}^{-1.209}\times 0.993^{\text{age}}\times 1.049. \end{array}$$

∗chronic kidney disease epidemiology collaboration in Asians (CKD-API-Asia)

### 2.3. Statistical Methods

Data analysis was carried out using SPSS 19.0 statistical software. The measurement data conforming to the normal distribution was expressed as mean ± standard deviation (*x* ± *s*) and *t*-test was used to compare the two groups. The measurement data that did not conform to the normal distribution was expressed as median ± four percentile and comparison between groups was performed by the Wilcoxon rank test. The counting data was expressed as relative numbers, and *X*^2^ test was used to compare the two groups. Repeated measurement variance analysis was used to analyze the repeated measurement data. *p* < 0.05 indicated that the difference was statistically significant.

## 3. Results

### 3.1. Baseline Comparison of the Two Groups

As shown in Table 1, there is no significant difference (*p* > 0.05) was between the two groups for the following characteristics: gender, age, educational level, disease duration, BMI, FBG, HbA1c, TG, TC, LDL-C, HDL-C, UA, BUN, and eGFR.

### 3.2. Comparison of Blood Glucose Level between Two Groups before and after Intervention

**Figure 1:**
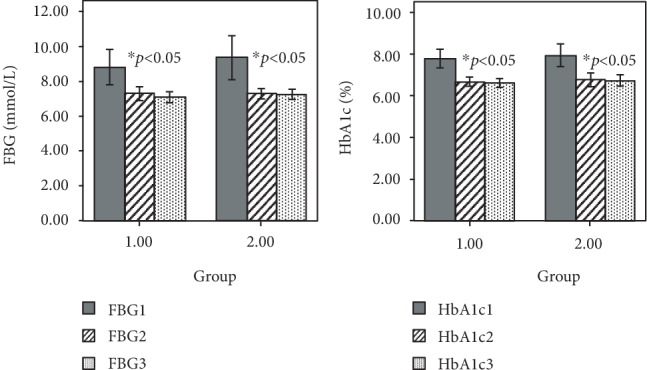
Comparison of FBG and HbA1c levels between the two groups after 3 months and 6 months of intervention. HbA1c1 is for the baseline of HbA1c, HbA1c2 is for the level of HbA1c after 3 months intervention, and HbA1c3 is for the level of HbA1c after 6 months intervention. FBG1 is for the baseline of FBG, FBG2 is for the level of FBG after 3 months intervention, and FBG3 is for the level of FBG after 6 months intervention. ^∗^*p*: compared with baseline,^∗∗^*p*: compared with indexes of 3 months.

**Figure 2:**
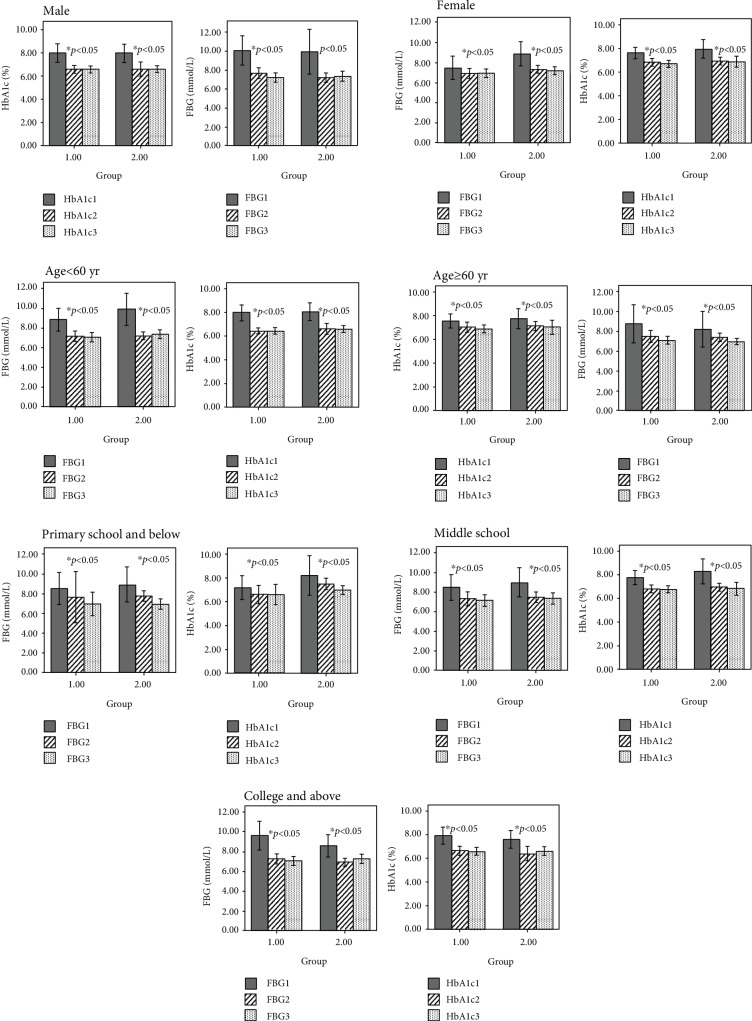
Comparison of FBG and HbA1c levels between the two groups after stratify age, sex and education level after 3 months and 6 months of intervention. HbA1c1 is for the baseline of HbA1c, HbA1c2 is for the level of HbA1c after 3 months intervention, and HbA1c3 is for the level of HbA1c after 6 months intervention. FBG1 is for the baseline of FBG, FBG2 is for the level of FBG after 3 months intervention, and FBG3 is for the level of FBG after 6 months intervention. ^∗^*p*: compared with baseline, ^∗∗^*p*: compared with indexes of 3 months.

The FBG and HbA1c levels in the two groups were significantly lower than those in the baseline after 3 months of intervention (*F* = 21.33, *p* < 0.05; *F* = 39.27, *p* < 0.05). With the prolongation of time (after 6 months), there was no significant change in the levels of FBG and HbA1c in the two groups (*F* = 0.38, *p* = 0.68; *F* = 0.05, *p* = 0.97). At the same time, there was no significant difference in decreasing FBG and HbA1c levels between the two method education (*F* = 0.54, *p* = 0.46; *F* = 0.42, *p* = 0.52), as shown in Table 2 and Figure 1. Further, after stratification of age, sex, and education, it showed that the FBG and HbA1c levels in the two groups were significantly lower than those in the baseline after 3 months of intervention (*p* < 0.05). However, after 6 months, there was no significant change between the FBG and HbA1c levels in the two groups (*p* > 0.05). At the same time, there was no significant difference in decreasing FBG and HbA1c levels between the two methods of education (*p* > 0.05), as shown in Table 3 and Figure 2.

### 3.3. Comparison of Blood Pressure and Lipid Levels between the Two Groups before and after Intervention

**Figure 3:**
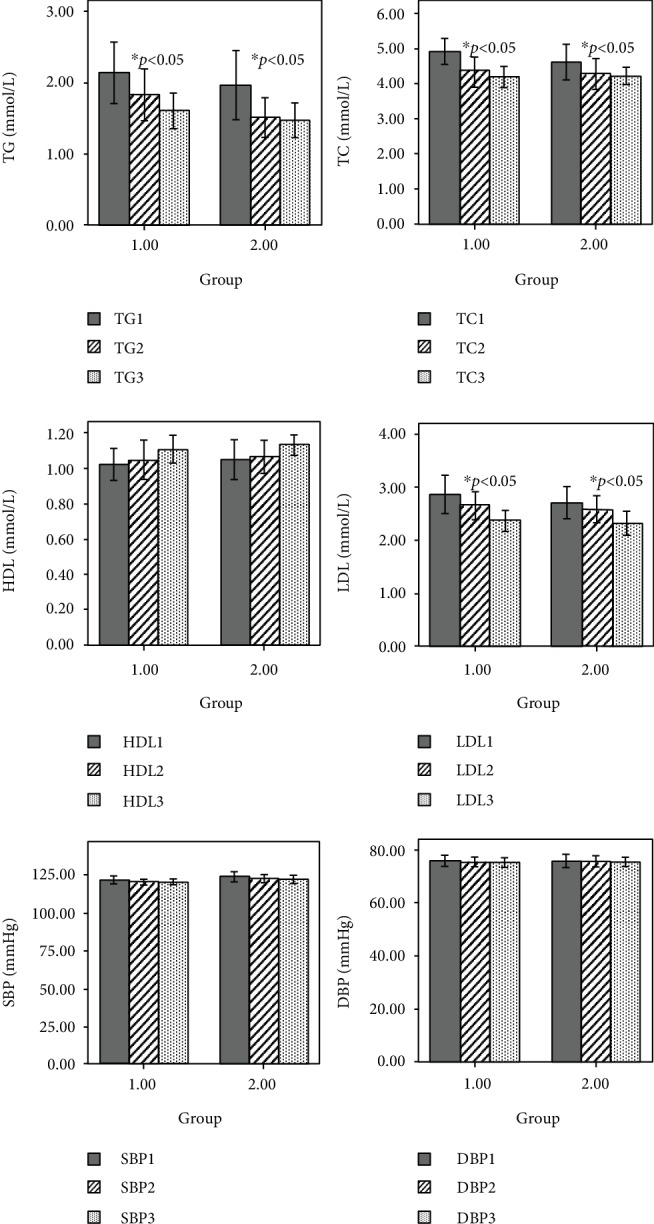
Comparison of BP and lipid levels 3 months and 6 months after intervention between the two groups. TC1 is for the baseline of TC, TC2 is for the level of TC after 3 months intervention, and TC3 is for the level of TC after 6 months intervention. TG1 is for the baseline of TG, TG2 is for the level of TG after 3 months intervention, and TG3 is for the level of TG after 6 months intervention. LDL-C1 is for the baseline of LDL-C, LDL-C2 is for the level of LDL-C after 3 months intervention, and LDL-C3 is for the level of LDL-C after 6 months intervention. HDL-C1 is for the baseline of HDL-C, HDL-C2 is for the level of HDL-C after 3 months intervention, and HDL-C3 is for the level of HDL-C after 6 months intervention. SBP1 is for the baseline of SBP, SBP2 is for the level of SBP after 3 months intervention, and SBP3 is for the level of SBP after 6 months intervention. DBP1 is for the baseline of DBP, DBP2 is for the level of DBP after 3 months intervention, and DBP3 is for the level of DBP after 6 months intervention. ^∗^*p*: compared with baseline.

Compared with the baseline blood pressure level, there was no significant change in the levels of SBP and DBP after 3 months and 6 months of intervention (*p* > 0.05). Compared with the baseline, the levels of TC, TG, and LDL-C in the two groups decreased significantly after 3 months of intervention (*F* = 5.727, *p* < 0.05; *F* = 6.47, *p* < 0.05; *F* = 6.57, *p* < 0.05); but with the prolongation of time, after 6 months, there was no significant change in these levels for the two groups (*p* > 0.05). At the same time, there was no significant difference in decreasing TC, TG, and LDL-C levels between the two methods of education (*p* > 0.05), as shown in Table 2 and Figure 3.

### 3.4. Comparison of Scales between the Two Groups before and after Intervention

**Figure 4:**
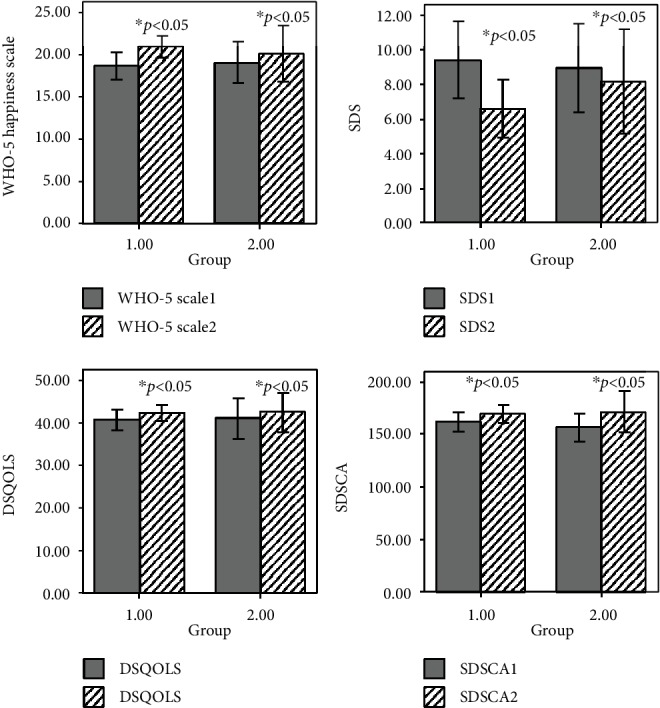
Comparison of WHO-5 Happiness Scale, SDS scale, DSQOLS scale, and SDSCA scale 3 months after intervention between the two groups. WHO-5 Scale1 is for the baseline of WHO-5 Scale1, and WHO-5 Scale12 is for the level of WHO-5 Scale after 3 months intervention. SDS 1 is for the baseline of SDS, and SDS2 is for the level of SDS after 3 months intervention. DSQOLS 1 is for the baseline of DSQOLS, and DSQOLS2 is for the level of DSQOLS after 3 months intervention. SDSCA1 is for the baseline of SDSCA, and SDSCA2 is for the level of SDSCA after 3 months intervention. ^∗^*p*: compared with baseline.

Compared with the baseline, there was a significant change in levels of WHO-5 Happiness Scale, SDS scale, DSQOLS scale, and SDSCA scale in the two groups after 3 months of intervention (*t* = −38.66, *p* < 0.05; *t* = 9.75, *p* < 0.05; *t* = −47.92, *p* < 0.05; *t* = 44.73, *p* < 0.05). However, there was no significant difference in change of WHO-5 Happiness Scale, SDS scale, DSQOLS scale and SDSCA scale levels after intervention (*p* > 0.05), as shown in Table 2 and Figure 4.

## 4. Discussion

The management of diabetic patients includes not only routine medical treatment but also health education to maintain psychological well-being.

Currently, the educational intervention of diabetes in China and around the world is mainly prescriptions and lifestyle restrictions from a medical staff. This is a passive transfer of theoretical knowledge, which lacks the patient's feedback. Although this improves the patient's condition, it does not achieve long-term results [6]. As a new method of chronic disease education, peer education is earning its place and recognition by clinical medical staff. At present, the impact of peer support education on blood glucose levels, HbA1c, and self-management ability of type 2 diabetic patients has been fully affirmed, resulting in guidelines for nursing of type 2 diabetes patients [7–9]. A meta-analysis [10] was conducted on the influence of peer education on HbA1c and self-management ability of type 2 diabetes patients in China. Subgroup analysis showed that compared with the control group, peer education that lasted less than 6 months and over 6 months could reduce the levels of HbA1c (*p* < 0.05). On the other hand, another meta-analysis showed that, even after long-term peer education (over 6 months and 12 months), there was no significant difference in HbA1c levels between the intervention and control groups.

The peer support education model works on the theory of incremental changes [6]. Initially, educators or nurses with professional knowledge train diabetic patients to be the leader of a peer support group. Then, the peer group leaders educate the patients around them. Due to an additional emotional bond over their similar situations, they also share information and experiences that they would normally abstain from sharing with medical workers. Listening, communicating, and learning from each other can help alleviate the psychological pressure caused by the disease and improve their quality of life.

The results of our study showed that after 3 months of either medical and peer support education, both the FBG and HbA1c levels of the patients were significantly lower than those of the baseline. This improvement unfortunately did not decrease along with time. Our results showed that after 6 months, there was no significant changes in the levels of FBG and HbA1c levels compared with those after 3 months of intervention. The reason may be due to the fact that after 3 months of intervention, the FBG and HbA1c levels of the subjects had been significantly improved, and HbA1c levels had dropped to less than 7%. Therefore, the lack of change after 6 months intervention could be due to the fact that the blood glucose level of the subjects had been stabilized instead of further decreasing.

After stratification of age, sex, and education level, the results still showed that the FBG and HbA1c of the patients decreased significantly after 3 months, but did not decrease significantly after 6 months (when compared with 3rd month reading). In addition, we also compared the level of blood sugar decline between the two education methods. It was found that after 3 months and 6 months, there is no significant difference of FBG and HbA1c between the two groups.

In 2007, the World Health Organization (WHO) initiated the “Peer Support Programmers in Diabetes” (DPSP) to bring together patients with similar life experiences and no hierarchy, to listen to and support each other, to discuss problems, and to share knowledge and experiences of one living with diabetes that most medical staff do not have [11]. The significance of peer support education includes (1) it can help diabetic patients have a spiritual resonance instead of a lonely struggle [12]; (2) diabetic patients require strict diets with a timed medication schedule. This may be easier to follow with peer reminders [13]; (3) group activities can help diabetic patients socialize, learn from, and help each other; (4) peer education is a resource that provides intimate medical and nursing services to patients [14]. Through the implementation of peer support education, systematic, regular, and continuous monitoring of patients can be carried out. This can help prevent chronic complications associated with diabetes, thereby improving their quality of life. Therefore, peer education model caters to both the psychological and physical well-being of diabetic patients. However, at present, there are limited reports available, both in China and around the world, about whether the peer support education model can replace the education of a medical staff in improving blood glucose and lipid levels for patients with uncontrolled blood glucose levels.

In our study, 120 subjects were chosen and the average level of HbA1c was less than 8.5%. Sixty pairs of peer education groups were formed. One group received direct education from the medical staff and the other group received peer support education. While the results showed an improvement in the blood glucose and lipid levels of the two groups when compared to initial levels, there was no significant level difference between the methods of education. The patients were observed for 6 months and there were no significant differences between the levels taken after the three and six months, indicating stable levels. This suggests that peer support education can replace the health education given by the medical staff in mild-moderate hyperglycemia diabetic patients.

There are some limitations in this study: (1) The number of samples needs to be expanded further in order to investigate the effect of peer support education model on the levels of glucose and lipids in patients with T2DM in China. (2) The blood glucose value of the subjects included in this study were not very high (HbA1c < 8.5%), so whether the peer support education model has the same effect is unknown. However, for the patients with severe hyperglycemia, medical intervention and drug adjustment would be needed rather than peer support education.

Both in China and around the world, peer support education for diabetes mellitus faces a lot of challenges. Despite this, we firmly believe that peer support education would benefit more patients, through the establishment of more glucose clubs and the inclusion of more volunteers [15]. From the results of this study, we believe that peer education can effectively decrease blood glucose levels and improve lipid metabolic indicators for mild-moderate hyperglycemia patients. This can help relieve the burden of the medical staff as well as help the patients to remain comfortable with their peers.

## Figures and Tables

**Table 1:** Baseline comparison of the two groups of subjects.

Index	Group 1	Group 2	*t*(*X*^2^)	*p*
Sex (male/female)	34/26	30/30	0.37^a^	0.55
Age (year)	52.88 ± 12.92	52.05 ± 12.73	0.36	0.72
Education level (%)				
Primary school and below	4 (6.7)	5 (8.3)		
Middle school	11 (18.3)	18 (30)		
College and above	45 (75.0)	37 (61.7)	2.61^a^	0.57
Duration (year)	6.79 ± 6.35	7.10 ± 7.36	-1.07	0.30
SBP (mmHg)	121.93 ± 9.36	123.64 ± 12.01	0.14	0.89
DBP (mmHg)	75.95 ± 8.07	75.75 ± 9.32	0.62	0.54
BMI (kg/m^2^)	25.48 ± 3.23	25.01 ± 3.13	1.31	0.19
FBG (mmol/L)	8.81 ± 3.89	9.36 ± 4.79	-0.83	0.41
HbA1c (%)	7.81 ± 1.77	7.97 ± 2.13	-0.60	0.55
TC (mmol/L)	4.92 ± 1.39	4.61 ± 1.73	0.83	0.41
TG (mmol/L)	2.14 ± 1.56	1.96 ± 1.58	0.10	0.92
LDL-C (mmol/L)	2.86 ± 1.31	2.70 ± 0.99	0.10	0.92
HDL-C (mmol/L)	1.02 ± 0.32	1.05 ± 0.40	-0.02	0.99
UA (mmol/L)	328.36 ± 92.24	342.14 ± 92.35	-0.74	0.46
BUN (mmol/L)	5.95 ± 6.38	5.73 ± 4.01	0.21	0.84
eGFR (ml/min/1.73^2^)	103.31 ± 14.08	99.14 ± 25.55	0.96	0.34
WHO-5 happiness scale	18.78 ± 5.54	18.26 ± 4.58	0.54	0.59
SDS	9.25 ± 6.93	10.08 ± 6.33	-0.66	0.51
DSQOLS	39.88 ± 8.44	39.66 ± 8.36	0.14	0.89
SDSCA	163.71 ± 24.31	159.49 ± 26.80	0.83	0.41

Note: a is for *X*^2^ value; BMI: body mass index; WHR: waist-to-hip ratio; SBP: systolic blood pressure; DBP: diastolic blood pressure; PP: pulse pressure; FBG: fasting blood glucose; HbA1c: glycosylated hemoglobin; UA: uric acid; TC: total cholesterol; TG: triglycerides; LDL-C: low-density lipoprotein cholesterol; HDL-C: high-density lipoprotein cholesterol; eGFR: estimated glomerular filtration rate; BUN: urea nitrogen;; SDS, Self-rating Depression Scale; DSQOLS, Diabetes-Specific Quality of Life Scale; SDSCA, Summary of Diabetes Self-Care Activities.

**Table 2:** Comparison of FBG and HbA1c levels between the two groups after 3 months and 6 months of intervention.

		Group 1			Group 2		*F*(*t*)	*p*
Index	Baseline	3 months	6 months	Baseline	3 months	6 months		
BMI	25.48 ± 3.23	25.22 ± 3.06	25.14 ± 2.99	25.01 ± 3.13	24.87 ± 2.93	24.78 ± 2.83	0.41	0.52
FBG	8.81 ± 3.89	7.30 ± 1.46	7.08 ± 1.21	9.36 ± 4.79	7.27 ± 1.11	7.26 ± 1.18	0.54	0.46
HbA1c	7.81 ± 1.77	6.71 ± 0.91	6.65 ± 0.79	7.97 ± 2.13	6.79 ± 1.24	6.76 ± 1.01	0.42	0.52
SBP	121.93 ± 9.69	120.47 ± 7.32	120.58 ± 7.72	123.64 ± 12.01	122.62 ± 10.29	122.24 ± 10.22	1.51	0.22
DBP	75.95 ± 8.07	75.50 ± 6.80	75.33 ± 6.63	75.75 ± 9.32	75.58 ± 7.92	75.56 ± 5.76	0.00	0.97
TC	4.92 ± 1.39	4.38 ± 1.39	4.21 ± 1.09	4.61 ± 1.73	4.28 ± 1.51	4.22 ± 0.84	0.56	0.46
TG	2.14 ± 1.56	1.83 ± 1.32	1.61 ± 0.89	1.96 ± 1.58	1.51 ± 0.91	1.48 ± 0.78	1.31	0.26
LDL-C	2.86 ± 1.31	2.66 ± 0.96	2.37 ± 0.72	2.70 ± 0.99	2.58 ± 0.86	2.32 ± 0.74	0.49	0.49
HDL-C	1.02 ± 0.32	1.05 ± 0.39	1.11 ± 0.27	1.05 ± 0.40	1.06 ± 0.34	0.13 ± 0.20	0.00	0.99
WHO-5 Happiness Scale	18.78 ± 5.54	20.91 ± 4.10	—	18.26 ± 4.58	20.07 ± 6.01	—	0.60	0.55
SDS	9.25 ± 6.93	6.61 ± 5.47	—	10.08 ± 6.33	8.14 ± 5.23	—	0.92	0.36
DSQOLS	39.88 ± 8.44	42.44 ± 6.20	—	39.66 ± 8.36	42.40 ± 8.36	—	0.02	0.99
SDSCA	163.71 ± 24.31	169.11 ± 25.46	—	159.49 ± 26.80	171.18 ± 28.92	—	0.08	0.79

Note: WHR: waist-to-hip ratio; SBP: systolic blood pressure; DBP: diastolic blood pressure; PP: pulse pressure; FBG: fasting blood glucose; HbA1c: glycosylated hemoglobin; UA: uric acid; TC: total cholesterol; TG: triglycerides; LDL-C: low-density lipoprotein cholesterol; HDL-C: high-density lipoprotein cholesterol; SDS: Self-rating Depression Scale; DSQOLS: Diabetes-Specific Quality of Life Scale; SDSCA: Summary of Diabetes Self-Care Activities.

**Table 3:** Comparison of FBG and HbA1c levels between the two groups after stratify age, sex, and education level after 3 months and 6 months of intervention.

		Group1			Group2		F	*p*
Index	Baseline	3 months	6 months	Baseline	3 months	6 months		
HbA1c_Female_	7.61 ± 1.21	6.85 ± 0.89	6.71 ± 0.78	7.98 ± 2.22	6.95 ± 0.90	6.89 ± 1.18	0.75	0.39
HbA1c_Male_	7.99 ± 2.17	6.57 ± 0.93	6.58 ± 0.82	8.08 ± 2.11	6.60 ± 1.54	6.61 ± 0.73	0.02	0.96
HbA1c_<60 years old_	7.99 ± 1.95	6.46 ± 0.72	6.45 ± 0.75	8.14 ± 2.32	6.62 ± 1.38	6.62 ± 0.86	0.59	0.45
HbA1c_≥60 years old_	7.56 ± 1.47	7.04 ± 1.05	6.91 ± 0.79	7.77 ± 1.76	7.15 ± 0.79	7.05 ± 1.22	0.09	0.77
HbA1c_Primary school and below_	7.95 ± 0.93	6.63 ± 0.49	6.63 ± 0.54	8.23 ± 1.16	7.51 ± 0.64	7.00 ± 0.47	0.01	0.91
HbA1c_Middle school_	7.75 ± 1.38	6.83 ± 0.68	6.77 ± 0.64	8.30 ± 2.43	6.98 ± 0.81	6.83 ± 1.25	1.99	0.17
HbA1c_College and above_	7.91 ± 2.06	6.64 ± 1.08	6.56 ± 0.91	7.73 ± 1.93	6.39 ± 1.53	6.63 ± 0.89	0.02	0.88
FBG_Female_	7.47 ± 3.04	6.91 ± 1.30	6.93 ± 1.15	8.87 ± 3.35	7.32 ± 1.07	7.18 ± 1.05	0.80	0.38
FBG_Male_	10.06 ± 4.22	7.66 ± 1.53	7.21 ± 1.28	10.17 ± 5.01	7.21 ± 1.18	7.37 ± 1.32	0.04	0.85
FBG_<60 years old_	8.85 ± 3.34	7.15 ± 1.49	7.05 ± 1.36	9.86 ± 5.13	7.20 ± 1.20	7.40 ± 1.34	0.14	0.71
FBG_≥60 years old_	8.76 ± 4.63	7.50 ± 1.43	7.01 ± 1.00	8.21 ± 3.74	7.43 ± 0.90	6.99 ± 0.65	1.03	0.12
FBG_Primary school and below_	8.53 ± 1.03	7.63 ± 1.66	6.98 ± 0.77	8.91 ± 2.36	7.76 ± 0.69	6.98 ± 0.77	0.03	0.86
FBG_Middle school_	8.49 ± 2.78	7.26 ± 1.52	7.09 ± 1.19	8.99 ± 3.43	7.48 ± 1.24	7.36 ± 1.34	0.14	0.71
FBG_College and above_	9.65 ± 4.11	7.28 ± 1.45	7.08 ± 1.30	8.88 ± 3.19	6.93 ± 1.04	7.30 ± 1.17	0.14	0.71

Note: FBG: fasting blood glucose; HbA1c: glycosylated hemoglobin.

## Data Availability

The data used to support the findings of this study are available from the corresponding author upon request.
